# Multimorbidity and its socio-economic associations in community-dwelling older adults in rural Tanzania; a cross-sectional study

**DOI:** 10.1186/s12889-022-14340-0

**Published:** 2022-10-14

**Authors:** Emma Grace Lewis, William K. Gray, Richard Walker, Sarah Urasa, Miles Witham, Catherine Dotchin

**Affiliations:** 1 Faculty of Medical Sciences, Population Health Sciences Institute, Baddiley-Clark Building, Newcastle University, Richardson Road, Newcastle upon Tyne, NE2 4AX UK; 2 Northumbria Healthcare NHS Foundation Trust, North Tyneside General Hospital, North Shields, UK; 3 Kilimanjaro Christian Medical Centre, Moshi, Kilimanjaro Tanzania; 4 AGE Research Group, NIHR Newcastle Biomedical Research Centre, Translational Clinical Research Unit, Newcastle University and Newcastle Upon Tyne NHS Trust, Newcastle upon Tyne, UK

**Keywords:** Multimorbidity, Older people, Sub-Saharan Africa, Frailty

## Abstract

**Objectives:**

This paper aims to describe the prevalence and socio-economic associations with multimorbidity, by both self-report and clinical assessment/screening methods in community-dwelling older people living in rural Tanzania.

**Methods:**

A randomised frailty-weighted sample of non-institutionalised adults aged ≥ 60 years underwent comprehensive geriatric assessment and in-depth assessment. The comprehensive geriatric assessment consisted of a history and focused clinical examination. The in-depth assessment included standardised questionnaires, screening tools and blood pressure measurement. The prevalence of multimorbidity was calculated for self-report and non-self-reported methods (clinician diagnosis, screening tools and direct measurement). Multimorbidity was defined as having two or more conditions. The socio-demographic associations with multimorbidity were investigated by multiple logistic regression.

**Results:**

A sample of 235 adults participated in the study, selected from a screened sample of 1207. The median age was 74 years (range 60 to 110 inter-quartile range (IQR) 19) and 136 (57.8%) were women. Adjusting for frailty-weighting, the prevalence of self-reported multimorbidity was 26.1% (95% CI 16.7–35.4), and by clinical assessment/screening was 67.3% (95% CI 57.0–77.5). Adjusting for age, sex, education and frailty status, multimorbidity by self-report increased the odds of being financially dependent on others threefold (OR 3.3 [95% CI 1.4–7.8]), and of a household member reducing their paid employment nearly fourfold (OR 3.8. [95% CI 1.5–9.2]).

**Conclusions:**

Multimorbidity is prevalent in this rural lower-income African setting and is associated with evidence of household financial strain. Multimorbidity prevalence is higher when not reliant on self-reported methods, revealing that many conditions are underdiagnosed and undertreated.

**Supplementary Information:**

The online version contains supplementary material available at 10.1186/s12889-022-14340-0.

## Introduction

Multimorbidity, taken as the presence of two or more chronic conditions is common in low- and middle-income countries (LMICs), including African countries [1, 2]. In African countries, as elsewhere, multimorbidity prevalence increases with age, is higher among women, and is negatively associated with educational attainment [1]. Multimorbidity in the continent is of particular public health importance given the successes of becoming the fastest ageing world region [3], and the changing HIV epidemic, leading to a generation living and ageing with the condition [4]. The limited multimorbidity research from the continent that focuses on older adults has reported wide variance in prevalence estimations; from 65% in adults ≥ 60 years in Burkina Faso [5], to 2.5% for discordant multimorbidity in urban-dwelling adults ≥ 40 years in Tanzania [6]. Larger epidemiological studies have tended to rely on estimates of multimorbidity, based on participants’ self-report [6], while other studies have employed a combination of methods that have included direct testing, for example of blood pressure or blood glucose [5, 7]. Multimorbidity across LMICs has tended to be positively associated with age, and lower socio-economic groups [8], however patterns have differed in areas of high HIV prevalence [7]. Outcomes associated with multimorbidity in LMICs include reduced quality of life, difficulty with the Activities of Daily Living (ADLs) and depression [8]. Multimorbidity has also been shown to impact on hospitalisation and healthcare costs, including out-of-pocket expenditure [6, 9, 10]. The concept of “geriatric syndromes” has long been embraced by geriatricians in high-income countries and is used to describe the common clinical conditions of older people with frailty, such as incontinence, falls, and delirium [11]. These problems have been rarely investigated in research of older people in African countries [12–14].

Overall, there is a stark imbalance between the prevalence of multimorbidity in LMICs and the region’s research output on the topic [15, 16]. This study aimed to address three research aims: First, to investigate the prevalence of multimorbidity by two different methods of data collection, allowing comparison between self-report and clinical assessment. Secondly, to explore the prevalence of geriatric syndromes, and their contribution to multimorbidity in this population, and thirdly, to examine the associations between multimorbidity and socio-economic characteristics in this setting.

## Methods

### Ethics and consent

Ethical approval was granted by two local ethics committees; the National Institute of Medical Research and Kilimanjaro Christian Medical University College Research Ethics Committee in Tanzania, and Newcastle University Research Ethics Committee in the UK. Verbal and written information was given to participants and their close relatives regarding the study, and the implications of taking part. A consent form was read aloud and discussed, to overcome difficulties in reading, either due to low educational attainment, poor vision or cognitive impairment. Consent forms were completed by signature or thumbprint, depending on literacy status. Where participants were unable to consent, assent was obtained from a close family member.

### Setting, recruitment and timing

Cross-sectional data were collected between 24^th^ February and 9^th^ August 2017 in the Hai district demographic surveillance site (DSS), located in the Kilimanjaro region of Northern Tanzania. Five villages were randomly selected. From within these villages census enumerators were asked to identify all adults aged ≥ 60 years. This list was cross-checked with the most recent census (2012), and with the village chairman and other community leaders, and refined to produce a denominator population for each village. All names listed were invited to participate in the study.

### Data collection methods

Data were collected on hand-held tablet computers using data collection forms developed in Open Data Kit (ODK) software. Data were uploaded daily to a secure encrypted server. Data collection started with recruiting and screening the denominator population of adults aged ≥ 60 years living in the five randomly selected villages using the “Brief Frailty Instrument for Tanzania” (B-FIT) [17]. A frailty-weighted randomisation procedure was then conducted using a random number list [18]. All participants who were found to be frail by the B-FIT screen (scoring 5–6), and a random sample of approximately 50% of pre-frail participants (scoring 2–4) and a random sample of approximately 10% of non-frail participants (scoring 0–1), were selected and invited for Comprehensive Geriatric Assessment (CGA) and in-depth assessment. This method of weighted randomisation has been used by our team to estimate the prevalence of dementia in the same region and was used given that the primary aim of the overall study was to investigate frailty prevalence [18, 19]. The current study was part of a wider study of frailty in the Hai district. The sample size was based on validation of the B-FIT frailty screen. We wished to assess the performance of the B-FIT with a standard error of no more than 0.03 (95% CI ± 0.65) and were seeking an AUROC of no less than 0.8, thus we aimed to recruit a minimum sample of 230 people.

Details regarding the procedures undertaken in performing the CGA and in-depth assessments have been previously published [18, 20]. The CGA was conducted by a UK-based clinician with experience of geriatrics and global health work, alongside a Tanzanian clinical officer or junior doctor. The assessment included a thorough history of the participant’s current physical symptoms and their past medical history. Where relevant, a collateral history was gained, particularly if cognitive or sensory impairment made this necessary. All participants underwent a physical examination, the nature of which was dependant on the participant’s history. This allowed the assessing clinicians to make a diagnosis of frailty, or not, and to formulate a list of probable diagnoses, independent of whether the participant had previously been given a diagnosis. In order to reduce the impact of confirmation bias, the clinicians were blind to the participant’s responses to the self-reported diagnoses. This list of probable diagnoses was then categorised by body system or disease category.

A separate in-depth assessment was carried out by trained local researchers. A series of standardised questionnaires were conducted alongside physical measurements detailed below:

### Self-reported diagnoses

Participants were asked “Have you ever been told you have a diagnosis of any of the following?”, a question taken from the Study of Global Ageing and Adult Health (SAGE) Questionnaire [21]. Seventeen different health conditions were listed, in order to include conditions affecting a breadth of body systems. Local Kiswahili expressions were used to improve lay understanding, for example, to refer to cataracts, the familiar expression *“ugonjwa wa mtoto wa jicho”* which literally translates as ‘the disease of child of the eye’, was used.

*Frailty syndromes:* Continence problems were derived from answers to the Barthel Index [22], and defined as requiring assistance with toileting or having at least occasional incontinence of bladder or bowel. Self-reported hearing difficulty was recorded based on an affirmative answer to the question “Do you think you have a hearing problem?”. The number of self-reported falls over the preceding year was recorded, where a fall was defined as *“unintentionally coming to rest on the floor, ground or other lower level”* [23].

*Mental health morbidity:* Cognition was assessed by the IDEA cognitive screen [24]. The following categorisations were used: 0–4 from a possible 12, indicating ‘probable dementia’, 5–7, ‘possible dementia’, 8–12, ‘no dementia’. Symptoms of depression were assessed using the EURO-D scale, with a total score of ≥ 5 indicative of depression [25, 26]. These validated screening tools do not confer a clinical diagnosis, but were used to diagnose probable cognitive impairment and/or depression as an alternative to self-report.

*Physical disability*: The Barthel Index [27], was used to grade an individual’s independence completing a range of Activities of Daily Living (ADLs) and mobility. The Barthel Index includes assessments of independence for activities such as dressing, toileting and grooming. ADL disability was classified as being unable to carry out any one of the activities independently.

### Operationalization of multimorbidity (including discordant multimorbidity)

*Self-reported multimorbidity*: The total number of self-reported health conditions (1. diabetes, 2. hypertension, 3. stroke, 4. cataracts, 5. arthritis, 6. heart disease, 7. respiratory disease, 8. Human immunodeficiency virus (HIV), 9. Tuberculosis (TB), 10. anaemia, 11. depression, 12. dementia, 13. (other) mental health condition, 14. gastro-intestinal disease, 15. epilepsy 16. cancer or 17. urological disease) were summed, with a possible range from 0 to 17. Self-reported multimorbidity was defined as reporting two or more health conditions. These 17 health conditions were assigned to one of the three multimorbidity domains: mental health (MH), non-communicable disease (NCD) and communicable disease categories (CD). The category CD included HIV and TB, while MH diagnoses were categorised as dementia, depression and other mental health conditions, all other conditions were assigned to NCDs.

*Non-self-reported multimorbidity*: The same diagnostic categories were formed from the documentation of the assessing clinician. Due to the limitations of making clinical diagnoses in these circumstances, without access to laboratory tests or psychiatric expertise, no diagnoses were made fitting the categories of ‘anaemia’ or ‘(other) mental health condition’. Rather, a category for ‘other’ diagnoses made clinically, such as orthostatic hypotension and essential tremor was included. (A full list of the ‘other clinical diagnoses’ is included in the supplemental material Table 1). Therefore, non-self-reported multimorbidity was calculated from a maximum of 16 possible health conditions.

**Table 1:** Demographic/socio-economic characteristics of the sample by sex

Demographic/health characteristic	Men *N* = 99 (%)	Women *N* = 136 (%)
Age category:		
60–69	38 (38.4)	49 (36.0)
70–79	28 (28.3)	40 (29.4)
≥ 80 years	33 (33.3)	47 (34.6)
Marital status:		
Married	68 (68.7)	43 (31.6)
Widowed	19 (19.2)	82 (60.3)
Separated/ divorced	10 (10.1)	9 (6.6)
Single (never married)	2 (2.0)	2 (1.5)
Education:		
University	5 (5.1)	3 (2.2)
Secondary school	5 (5.1)	3 (2.2)
Primary school	40 (40.4)	24 (17.6)
Some primary	36 (36.4)	48 (35.3)
No formal education	13 (13.1)	58 (42.6)
Literacy:		
Reads and/or writes easily	60 (60.6)	39 (28.7)
Reads and/or writes with difficulty	20 (20.2)	37 (27.2)
Not able to read and/or write	19 (19.2)	60 (44.1)
Lives alone	8 (8.2)	15 (11.1)
Pension	10 (10.1)	7 (5.1)
In the last 1 year, have any of your household members had to **reduce** their paid employment in order to spend time caring for your older relative?	17 (17.2)	30 (22.1)
In the last 1 year, have any of your household members had to **stop** their paid employment in order to spend time caring for your older relative?	7 (7.1)	17 (12.5)
Household difficulty paying school fees	8 (8.1)	18 (13.2)
CGA- diagnosed frailty:		
Frail	34 (34.3)	57 (41.9)
Not frail	65 (65.7)	79 (58.1)
CASP-19 (Mean 24.48, range 0–53, SD 11.63)		
Mean (SD)	22.38 (12.5)	26.0 (10.7)
ADL disability:		
Difficulty with ≥ 1 ADLs	26 (26.3)	57 (41.9)
Non-self-reported MH multimorbidity:		
None	61 (61.6)	53 (39.0)
Depression or Dementia	31 (31.3)	64 (47.1)
Depression and Dementia	7 (7.1)	19 (14.0)
Self-reported mental health multimorbidity:		
None	90 (90.9)	116 (85.3)
Depression or Dementia (or other MH diagnosis)	9 (9.1)	16 (11.8)
Depression and Dementia (or other MH diagnosis)	0 (0.0)	4 (2.9)
Non-self-reported diagnoses: (from 16)^*^		
None	13 (13.1)	7 (5.1)
1 diagnosis	21 (21.2)	20 (14.7)
2 diagnoses	26 (26.3)	31 (22.8)
3 diagnoses	24 (24.2)	39 (28.7)
4 diagnoses	12 (12.1)	26 (19.1)
5 diagnoses	3 (3.0)	9 (6.6)
6 diagnoses	0 (0.0)	4 (2.9)
Self-reported diagnoses: (from 17)^**^		
None	39 (39.4)	41 (30.1)
1 diagnosis	36 (36.4)	42 (30.9)
2 diagnoses	8 (8.1)	29 (21.3)
3 diagnoses	13 (13.1)	11 (8.1)
4 diagnoses	3 (3.0)	5 (3.7)
5 diagnoses	0	5 (3.7)
6 diagnoses	0	2 (1.5)
7 diagnoses	0	1 (0.7)
Non-self-reported NCD multimorbidity (from 12)		
1 NCD condition	34 (34.3)	36 (26.5)
2 NCD condition	29 (29.3)	46 (33.8)
≥ 3 NCD condition	21 (21.2)	42 (30.9)
Self-reported NCD multimorbidity (from 12 excluding depression, dementia and other mental health disorders)		
1 NCD condition	33 (33.3)	42 (30.9)
2 NCD conditions	14 (14.1)	29 (21.3)
≥ 3 NCD conditions	10 (10.1)	19 (14.0)
Non-self-reported CD		
None	95 (96.0)	132 (97.1)
TB or HIV	4 (4.0)	4 (2.9)
TB and HIV	0	0
Self-reported CD		
None	96 (97.0)	133 (97.8)
TB or HIV	3 (3.0)	3 (2.2)
TB and HIV	0	0
Geriatric multimorbidity^***^:		
None	28 (28.3)	27 (19.9)
One	28 (28.3)	34 (25.0)
Two	21 (21.2)	41 (30.1)
Three	20 (20.2)	24 (17.6)
Four	0	9 (6.6)
Five	2 (2.0)	1 (0.7)
Non-self-reported ‘discordant’ multimorbidity:		
NCD or CD or MH diagnosis	47 (47.5)	48 (35.3)
NCD *and/or* MH *and/or* CD diagnoses	38 (38.4)	80 (58.8)
NCD *and* MH *and* CD diagnoses	1 (1.0)	1 (0.7)
Self-reported ‘discordant’ multimorbidity:		
NCD or CD or MH diagnosis	51 (51.5)	77 (56.6)
NCD *and/or* MH *and/or* CD diagnoses	9 (9.1)	18 (13.2)

^*^Depression by EURO-D (using cut off ≥ 5/12 for probable depression), cognitive impairment by IDEA tool (IDEA screening tool ≤ 4/12), hypertension (recorded when average systolic BP and/or diastolic BP were elevated (Systolic BP ≥ 140 mmHg and/or diastolic BP ≥ 90 mmHg) and the following diagnostic categories; epilepsy, cancer, urological, HIV, TB, arthritis, respiratory disease, heart disease, gastro-intestinal conditions, stroke, cataracts, diabetes and other diagnoses

^**^The number of chronic diseases was derived from self-reported diagnoses of any of the following; (diabetes, hypertension, stroke, cataracts, arthritis, heart disease, respiratory disease, HIV, TB, anaemia, depression, dementia, other mental health conditions, gastro-intestinal conditions, epilepsy, cancer, urological conditions)

^***^Geriatric multimorbidity ≥ 2 of the following: ≥ 2 falls in the previous year, continence problems, self-reported hearing difficulty, CGA-diagnosed cataracts or arthritis and cognitive impairment by the IDEA tool

*Discordant multimorbidity*: The total number of domains (from CD, NCD and MH) with at least one condition present were summed, with a possible range from 1 to 3. Discordant multimorbidity was defined as having at least one condition in two or more health domains.

*Geriatric multimorbidity*: In order to encompass the common ‘frailty syndromes’ [28], ‘geriatric multimorbidity’ was defined as ≥ 2 of the following: ≥ 2 falls in the previous year (by self-report), continence problems (derived from answers to the Barthel Index), self-reported hearing difficulty, CGA-diagnosed cataracts, CGA-diagnosed arthritis and cognitive impairment by the IDEA screen.

*Self-reported quality of life:* the CASP-19 scale, which has been used widely, including in African settings, [29] was translated into Kiswahili by a qualified linguist and back-translated to ensure equivalence of meaning. The CASP-19 scores were calculated as per standard recommendations producing a score between 0–57 [30]. Mean and standard deviations for CASP-19 scores have been presented by socio-demographic or health characteristic and for the multiple regression analysis, a categorical variable was produced, dividing the score distribution by quartiles.

### Blood pressure measurement

Blood pressure (BP) was measured three times in the participant’s right arm with the participant sitting, using an A&D Medical UA-704 digital blood pressure monitor. High BP was categorised by an elevated average systolic BP and/or diastolic BP (Systolic BP ≥ 140 mmHg and/or diastolic BP ≥ 90 mmHg). While a single episode of BP measurement would be inadequate to make a clinical diagnosis of hypertension, for the purposes of this study a high average BP was categorised as hypertension by non-self-report.

### Socio-economic factors

In order to examine the impact of the older person’s multimorbidity on the household’s finances, the participant or their close relative was asked; *‘Are you/Is the participant completely financially/materially dependent on family?’*, if no, we recorded whether they were in receipt of a pension. The following question aimed to gauge the opportunity-cost of multimorbidity on households; *‘In the past year, have any of your household members had to reduce their paid employment in order to spend time caring for you/r older relative?’*. In order to investigate the impact of multimorbidity on the household’s competing expenditures we asked *‘In the last 1 year, has the cost of healthcare for the participant affected the ability to pay for other things like school fees?’*.

### Statistical analysis

Statistical analyses were supported by IBM SPSS for Windows version 26 (IBM Corp, Armonk, NY, USA) and StataIC 16 (64-bit) software. Descriptive statistical analysis used standard summary measures depending on the nature of the data. Descriptive data were presented by sex, as being female, of a low socio-economic status and low educational attainment are all known risk-factors for multimorbidity [16]. When calculating the prevalence of self- and non-self-reported conditions, the random frailty-weighted stratification (based on B-FIT score) was taken into account using inverse proportions (described in the methods section and published in detail previously [18]). To calculate confidence intervals, bootstrapping (Stata command ‘svyset’) was used to control for clustering by village and to adjust for the stratified weighting [18]. Proportional Venn diagrams were used (Stata command ‘pvenn’) to illustrate the comparative size and overlap of ‘discordant’ multimorbidity. In order to compare means in CASP-19 scores, independent t-testing was used for binary variables and one way ANOVA for categorical variables. Multiple regression analysis of variables associated with multimorbidity used odds ratios (ORs) with 95% confidence intervals (CIs). Significance was assumed at the 5% level. There were few missing values, except for the CASP-19 where four participants (1.7%) failed to complete the questionnaire and data were analysed for the complete questionnaires (*N *= 231). For the categorical variables where one or two data points were missing (lives alone, and health insurance), these were imputed using zero or constant imputation.

## Results

A total of 1,207 participants underwent screening, this accounted for between 84.5% and 89.0% of eligible participants in each village [18]. Following randomisation, 236 were selected to receive CGA and in-depth assessments. The flow diagram for recruitment has been published previously [18]. Data from 235 individuals were included in this analysis as one participant withdrew from the study after their CGA. The median age was 74 years (range 60 to 110, IQR 19) and 136 (57.8%) were women. Demographic and socio-economic characteristics of the frailty-weighted sample by sex revealed that 60.3% of women were widowed, while 68.7% of men remained married. Almost half of the women had received no formal education and were illiterate, while the majority of men had attended or completed primary school. A minority lived alone or were in receipt of a pension, see Table 1.

**Fig. 1:**
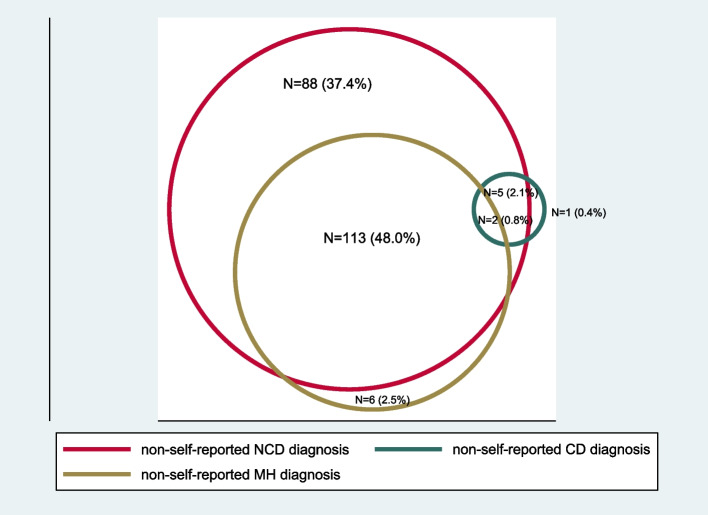
Non-self-reported discordant multimorbidity

**Fig. 2:**
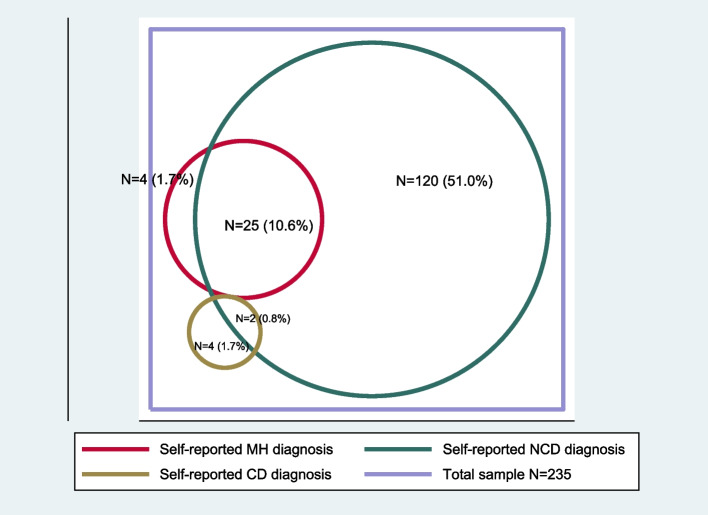
Self-reported discordant multimorbidity

When adjusted for frailty-weighting, the prevalence of self-reported multimorbidity was 26.1% (95% CI 16.7–35.4), and by clinical assessment/screening it was 67.3% (95% CI 57.0–77.5), see Table 2. For all health conditions, except diabetes, the adjusted prevalence was higher when the diagnosis was based on non-self-reported methods, rather than self-report (supplemental material Fig. 1). The adjusted prevalence of the experimental construct ‘geriatric multimorbidity’ was 34.9% (95% CI 29.3–40.5). Multimorbidity was associated with higher odds of having difficulty with one or more ADLs, and with poorer CASP-19 quality of life scores, yet significance was lost after adjusting for age, sex, education and frailty status (Table 3). A three-fold increased odds of CGA frailty was found in those with multimorbidity (after adjustment). When adjusting for age, sex, education and frailty status, multimorbidity by self-report increased the odds of being financially dependent on others threefold (OR 3.3 [95% CI 1.4–7.8]), and of a household member reducing their paid employment nearly fourfold (OR 3.8. [95% CI 1.5–9.2]) (Table 3). Figures 1 and 2 illustrate the size and proportional overlap in each domain, producing discordant multimorbidity between CD, NCD and MH domains.

**Table 2:** The adjusted prevalence of multimorbidity

Condition/type of multimorbidity	Self-report N from 235 (%)	Self-report adjusted prevalence (95% CI)	Clinical assessment N from 235 (%)	Clinical assessment adjusted prevalence (95% CI)
Multimorbidity	77 (32.8)	26.09 (16.7–35.5)	174 (74.0)	67.28 (57.1–77.5)
MH multimorbidity	4 (1.7)	0.57 (-0.4–1.6)	26 (11.1)	3.41 (2.3–4.6)
NCD multimorbidity	72 (30.6)	23.02 (15.6–30.4)	138 (58.7)	49.50 (41.6–57.4)
Discordant multimorbidity	27 (11.5)	9.58 (3.2–16.0)	120 (51.0)	40.81 (34.2–47.5)
Geriatric multimorbidity	118 (50.2)	34.88 (29.3–40.5)		

*MH* Mental health, *NCD* Non-communicable disease, *CI* Confidence interval

**Table 3:** The association between socio-demographic factors and self-reported multimorbidity

	No multimorbidity *N* = 158 (%)	Multimorbidity *N* = 77 (%)	Crude OR (95% CI) P value	Adjusted OR^*^ (95% CI) P value
CGA frailty (*n* = 91)	49 (31.0)	42 (54.5)	2.66 (1.4–4.7) *p* = 0.0005	3.05 (1.4–6.5) *p* = 0.002^*^
ADL disability (*n* = 83)	45 (28.4)	38 (49.3)	2.44 (1.3–4.3) *p* = 0.001	1.4 (0.6–3.0) *p* = 0.3
CASP-19 (*N* = 231) scores > 75^th^ percentile	33 (21.1)	26 (34.7)	1.9 (1.0–3.6) *p* = 0.02	1.4 (0.7–3.0) *p* = 0.3
CASP-19 (*N* = 231) Scores > 50^th^ percentile	72 (46.1)	48 (64.0)	2.0 (1.2–3.7) *p* = 0.01	1.5 (0.8–3.1) *p* = 0.2
Socio-economic factors				
No health insurance (*n* = 174)	117 (74.5)	57 (74.0)	0.9 (0.5–1.8) *p* = 0.93	1.0 (0.5–2.1) *p* = 0.9
Financially dependent (*n* = 104)	52 (32.9)	52 (67.5)	4.2 (2.2–7.8) *p* = < 0.0001	**3.3 (1.4–7.8) *p* = 0.002**
A household member has **reduced** their paid employment to provide care for the older person (*n* = 47)	19 (12.0)	28 (36.3)	4.1 (2.0–8.4) *p* = < 0.0001	**3.8 (1.5–9.2) *p* = 0.001**
A household member has **stopped** their paid employment to provide care for the older person (*n* = 24)	10 (6.3)	14 (18.1)	3.2 (1.3–7.9) *p* = 0.004	1.7 (0.6–4.7) *p* = 0.2
The cost of healthcare for the older person has affected the ability to pay for school fees (*n* = 26)	12 (7.5)	14 (18.1)	2.7 (1.1–6.2) *p* = 0.01	2.3 (0.9–5.8) *p* = 0.06

^*^Adjusted for age, sex, education status and CGA-diagnosed frailty, except for calculating the adjusted odds of multimorbidity in frailty. Results in bold indicate statistical significance

The proportional Venn diagrams included in the supplemental material (Figs. 2 and 3), illustrate that frailty, disability and multimorbidity are distinct and overlapping. CASP-19 scores were available for 231 of participants, (mean 24.5 SD 11.6 range 0–53). Univariate analysis showed significantly higher mean scores in women, older age groups, the frail and multimorbid (supplemental material Table 4).

## Discussion

### The prevalence of multimorbidity

Most of the large epidemiological studies of multimorbidity conducted in LMICs have been reliant on self-reported survey data and symptom-based questions combined with a diagnostic algorithm for conditions such as angina pectoris (employed in the World Health Organization’s multi-country Study on global AGEing and adult health (WHO SAGE)) [1, 8–10, 21]. Rarely have large community-based studies been able to conduct direct diagnostic testing, however when this has been achieved, a large burden of undiagnosed and untreated disease is revealed. For example, a cross-sectional study conducted in Malawi found 40% of people with diabetes were undiagnosed, and almost 60% with hypertension were unaware of their diagnosis [31]. Similarly, in a community study of adults aged ≥ 60 years in Burkina Faso, 42% of adults with hypertension and 21% of adults with diabetes received their diagnosis for the first time as study participants [5]. In this study, the adjusted prevalence of hypertension by self-report was 25.4% (95% CI 19.3–31.5), however, by direct measurement 48.1% (95% CI 38.4–57.8) were hypertensive (supplemental material Table 3). A similar pattern can be seen with almost every condition measured, which inevitably has an important impact on identifying and characterising the patterns of multimorbidity, particularly in settings of poorly resourced health systems. It may also explain some of the variance in multimorbidity prevalence estimates between studies: The prevalence of non-self-reported multimorbidity in this study was 67.3% (95% CI 57.0–77.5). This is much higher than the mean multimorbidity prevalence of 21.3% for those aged over 65 years from the World Health Survey [1]. Our study reports a multimorbidity prevalence similar to a comparative study conducted in Burkina Faso, that found 65% of their study participants had two or more chronic conditions [5]. This concordance is likely due to employing similar methods, combining questionnaires with a review of medical notes and clinical assessment for 389 adults aged ≥ 60 years. In a comparison between self-reported diagnoses of NCDs and criterion-based or symptom-based reporting, it was found that reliance on self-reported diagnoses tended to give rise to positive socio-economic group gradients, whereas symptom-based, or criterion-based measures tended to display less positive gradients or even negative gradients (with higher prevalence in lower socio-economic groups) [32]. These authors suggest reasons for this underestimation include a lack of access to diagnostics and healthcare services, a lack of awareness of NCDs, and low levels of literacy, all likely to be problems for rural-dwelling older people served by a poorly resourced healthcare system.

The prevalence of discordant multimorbidity was relatively low in this study, and CD multimorbidity was strikingly not found, such that the discordant multimorbidity was largely accounted for by MH and NCDs. A recent multimorbidity study in a large sample of adults aged ≥ 40 years (mean age 53) in the commercial capital of Tanzania, Dar es Salaam, found a quarter of their population had multimorbidity (defined as two or more conditions by self-report, from a total of eight) [6]. Theirs was a younger, more educated, and urban-dwelling population with higher rates of both HIV (5.2%) and TB (10.5%), but NCDs remained the most prevalent domain. The difference between this and our study’s CD prevalence is likely to reflect a lower regional prevalence. HIV prevalence in Kilimanjaro region is 2.6% for all adults above 15 years, but is lower in older age groups [33]. The relatively high prevalence of discordant multimorbidity (of NCD and MH conditions) is important, as it has previously been associated with poorer outcomes of greater frailty, and worse quality of life in a study of older adults in Burkina Faso [34].

### Geriatric multimorbidity

Patterns or clusters of multimorbidity have been described by three patterns of multimorbidity derived from a systematic review of the literature; either cardiovascular or metabolic diseases, mental health disorders and musculoskeletal conditions [35]. This study found that the construct of ‘geriatric multimorbidity’, inclusive of arthritis, dementia, incontinence, cataracts and falls, had an adjusted prevalence of around one third. There has been little research investigating syndromes associated with frailty in lower-income African settings, however a small cross-sectional study of people aged ≥ 60 years in Blantyre, Malawi, found a high proportion of those reporting falls in the previous year also reported memory problems and incontinence [12]. Analysis from the WHO SAGE study has shown that risk factors for falls-associated injury in LMICs include multimorbidity, depression, and cognitive impairment [36]. Further work should be undertaken to investigate this observed pattern of multimorbidity in lower-income African settings, which could help clarify whether the speculative term ‘geriatric multimorbidity’ is a helpful construct. In the authors’ view, quantifying the contribution of frailty syndromes to multimorbidity in older African populations may promote the development of better integrated geriatric healthcare, which falls far behind demand across the continent [37]. Tanzania’s national ageing policy recognises the difficulties facing older people, particularly living rurally, in accessing quality healthcare [38].

### Determinants of multimorbidity

Multiple regression analysis demonstrated that having multiple chronic medical problems is likely to put the household under financial strain, limiting both the individual with multimorbidity, and their family members’ ability to earn. In this setting, subsistence farming is the primary source of food and income, using manual farming methods on small family-owned plots of land. A minority of older people lived alone in this context. Often, older people were members of multi-generational households living with grandchildren, thus school fees were an important household expenditure. There is currently no universal state pension in Tanzania [39], and the minority in this study were in receipt of a pension. These findings are in line with the recent Dar es Salaam study, which found a significant association between multimorbidity and household food insecurity and hospitalisation, markers of both household financial strain and increased healthcare spending [6]. When comparing households affected by chronic illness (defined as any illness lasting ≥ 6 months), with households not affected by chronic illness in Tanzania, an increased out-of-pocket expenditure of 22% was found [40]. In a study of 1018 adults aged ≥ 60 in Tanzania, factors associated with out-of-pocket healthcare expenditure were visual impairment, functional disability, lack of formal education and traditional healer visits [41]. Secondary analysis of WHO SAGE data revealed that multimorbidity is associated with greater primary and secondary healthcare utilisation and consequent greater financial burden, driven in some cases by higher out-of-pocket expenditures, for example in order to purchase medicines [9]. This study’s findings highlight the need both for better integrated and more equitable healthcare, in order to address the healthcare needs of older people in lower-income settings.

### Strengths and limitations

The cross-sectional nature of this study means that causal inference is not possible, and the influence of reverse causality could account for some of the findings. However, our interpretations, for example of the socioeconomic factors, are resonant with other studies of the financial impact of multimorbidity in Tanzania and other LMIC countries [9, 41]. Some of the chronic conditions included as part of our multimorbidity condition list, have elsewhere been seen as outcomes of chronic multimorbidity, for example depression [8], however this is reflective of the heterogeneity of methods and definitions found in multimorbidity research [16].

Clinical diagnoses from the participant’s CGA were based on the history/collateral history and focused examination of the assessing clinician. This will have introduced certain biases, for example towards conditions with more evident physical signs, such as joint deformity in arthritic conditions, and away from diagnoses which require laboratory diagnostics, for example diabetes mellitus and anaemia. This bias, due to a lack of diagnostic testing, may have led to an under-estimation of the prevalence of multi-morbidity by clinical assessment, meaning that our finding is likely to be a conservative estimate.

This study is a valuable contribution to the limited research to date investigating the prevalence, pattern and associations with multimorbidity in older adults living in lower-income settings. There are very few studies which have succeeded in allowing a comparison between self-reported and alternative methods for identifying multimorbidity, especially while including such an extensive list of conditions, across CD, NCD and MH domains, and in such an understudied population. These findings suggest that under-diagnosis and consequent under-treatment are huge challenges facing lower-resourced health systems. The novel concept of ‘geriatric multimorbidity’ requires further investigation, but may be useful, particularly when seeking to develop integrated health services designed to address multimorbidity in older people.

## Conclusion

Multimorbidity is highly prevalent in this population, as is the underdiagnosis and undertreatment of many contributing conditions. Frailty syndromes were notably important to multimorbidity in this study and this is a topic ripe for further investigation and characterisation. Addressing the health challenges posed by multimorbidity in older African populations will require developing more integrated and accessible healthcare.

## Supplementary Information

**Additional file 1: Table 1. **List of conditions in the category ‘other clinical diagnoses’ by non-self-report.

**Additional file 2: Table 2. **Demographic/socio-economic characteristics of the sample by sex.

**Additional file 3: Table 3. **The prevalence of multimorbidity adjusted for frailty-weighting.

**Additional file 4: Table 4. **The CASP-19 quality of life score by demographic and multimorbidity categories.

**Additional file 5: Figure 1. **The adjusted prevalence of multimorbidity/conditions by self- and non-self-reported methods.

**Additional file 6: Figure 2. **The overlap between self-reported multimorbidity, disability and CGA-diagnosed frailty.

**Additional file 7: Figure 3. **The relationship between non-self-reported multimorbidity, CGA-diagnosed frailty and disability.

## Data Availability

The dataset generated and/or analysed during the current study are available in the Newcastle University data repository, [https://data.ncl.ac.uk/].

## References

[1] Afshar S, Roderick PJ, Kowal P, Dimitrov BD, Hill AG (2015). Multimorbidity and the inequalities of global ageing: a cross-sectional study of 28 countries using the world health surveys. BMC Public Health.

[2] Nguyen H, Manolova G, Daskalopoulou C, Vitoratou S, Prince M, Prina AM. Prevalence of multimorbidity in community settings: a systematic review and meta-analysis of observational studies. J Comorb. 2019;9:1–15. 10.1177/2235042X19870934 PMC6710708 31489279

[3] WHO: World Report on Ageing and Health. In. Geneva: World Health Organization; 2015. p. 267. https://www.who.int/publications/i/item/9789241565042. Accessed 14 Oct 2022.

[4] Negin J, Barnighausen T, Lundgren JD, Mills EJ (2012). Aging with HIV in Africa: the challenges of living longer. AIDS.

[5] Hien H, Berthe A, Drabo MK, Meda N, Konate B, Tou F, Badini-Kinda F, Macq J (2014). Prevalence and patterns of multimorbidity among the elderly in Burkina Faso: cross-sectional study. Trop Med Int Health.

[6] Tomita A, Leyna GH, Kim HY, Moodley Y, Mpolya E, Mogeni P, Cuadros DF, Dzomba A, Vandormael A, Barnighausen T (2021). Patterns of multimorbidity and their association with hospitalisation: a population-based study of older adults in urban Tanzania. Age Ageing.

[7] Chang AY, Gomez-Olive FX, Payne C, Rohr JK, Manne-Goehler J, Wade AN, Wagner RG, Montana L, Tollman S, Salomon JA (2019). Chronic multimorbidity among older adults in rural South Africa. BMJ Glob Health.

[8] Arokiasamy P, Uttamacharya U, Jain K, Biritwum RB, Yawson AE, Wu F, Guo Y, Maximova T, Espinoza BM, Rodriguez AS (2015). The impact of multimorbidity on adult physical and mental health in low- and middle-income countries: what does the study on global ageing and adult health (SAGE) reveal?. BMC Med.

[9] Lee JT, Hamid F, Pati S, Atun R, Millett C (2015). Impact of noncommunicable disease multimorbidity on healthcare utilisation and out-of-pocket expenditures in middle-income countries: cross sectional analysis. PLoS ONE.

[10] Sum G, Salisbury C, Koh GC, Atun R, Oldenburg B, McPake B, Vellakkal S, Lee JT (2019). Implications of multimorbidity patterns on health care utilisation and quality of life in middle-income countries: cross-sectional analysis. J Glob Health.

[11] Inouye SK, Studenski S, Tinetti ME, Kuchel GA (2007). Geriatric syndromes: clinical, research, and policy implications of a core geriatric concept. J Am Geriatr Soc.

[12] Allain TJ, Mwambelo M, Mdolo T, Mfune P (2014). Falls and other geriatric syndromes in Blantyre, Malawi: a community survey of older adults. Malawi Med J.

[13] Kalula SZ, Ferreira M, Swingler GH, Badri M (2016). Risk factors for falls in older adults in a South African urban community. BMC Geriatr.

[14] Lewis E, Paddick SM, Banks J, Duinmaijer A, Tucker L, Kisoli A, Cletus J, Lissu C, Dotchin C, Gray W (2016). Prevalence of delirium in older medical inpatients in Tanzania. J Am Geriatr Soc.

[15] Xu X, Mishra GD, Jones M (2017). Mapping the global research landscape and knowledge gaps on multimorbidity: a bibliometric study. J Glob Health.

[16] Xu X, Mishra GD, Jones M (2017). Evidence on multimorbidity from definition to intervention: an overview of systematic reviews. Ageing Res Rev.

[17] Gray WK, Orega G, Kisoli A, Rogathi J, Paddick SM, Longdon AR, Walker RW, Dewhurst F, Dewhurst M, Chaote P (2017). Identifying frailty and its outcomes in older people in rural Tanzania. Exp Aging Res.

[18] Lewis EG, Wood G, Howorth K, Shah B, Mulligan L, Kissima J, Dotchin C, Gray W, Urasa S, Walker R (2018). Prevalence of frailty in older community-dwelling Tanzanians according to comprehensive geriatric assessment. J Am Geriatr Soc.

[19] Longdon AR, Paddick SM, Kisoli A, Dotchin C, Gray WK, Dewhurst F, Chaote P, Teodorczuk A, Dewhurst M, Jusabani AM (2013). The prevalence of dementia in rural Tanzania: a cross-sectional community-based study. Int J Geriatr Psychiatry.

[20] Lewis EG, Whitton LA, Collin H, Urasa S, Howorth K, Walker RW, Dotchin C, Mulligan L, Shah B, Mohamed A (2020). A brief frailty screening tool in Tanzania: external validation and refinement of the B-FIT screen. Aging Clin Exp Res.

[21] Kowal P, Chatterji S, Naidoo N, Biritwum R, Fan W, Lopez Ridaura R, Maximova T, Arokiasamy P, Phaswana-Mafuya N, Williams S (2012). Data resource profile: the world health organization study on global AGEing and adult health (SAGE). Int J Epidemiol.

[22] Wade DT, Collin C (1988). The Barthel ADL Index: a standard measure of physical disability?. Int Disabil Stud.

[23] Kojima G, Kendrick D, Skelton DA, Morris RW, Gawler S, Iliffe S (2015). Frailty predicts short-term incidence of future falls among British community-dwelling older people: a prospective cohort study nested within a randomised controlled trial. BMC Geriatr.

[24] Gray WK, Paddick SM, Kisoli A, Dotchin CL, Longdon AR, Chaote P, Samuel M, Jusabani AM, Walker RW (2014). Development and validation of the identification and intervention for dementia in elderly Africans (IDEA) study dementia screening instrument. J Geriatr Psychiatry Neurol.

[25] Prince MJ, Reischies F, Beekman AT, Fuhrer R, Jonker C, Kivela SL, Lawlor BA, Lobo A, Magnusson H, Fichter M (1999). Development of the EURO-D scale–a European, union initiative to compare symptoms of depression in 14 European centres. Br J Psychiatry.

[26] Guerra M, Ferri C, Llibre J, Prina AM, Prince M (2015). Psychometric properties of EURO-D, a geriatric depression scale: a cross-cultural validation study. BMC Psychiatry.

[27] Mahoney FI, Barthel D (1965). Functional evaluation: The barthel index. Md State Med J.

[28] British Geriatrics Society, AgeUK, Royal College of General Practitioners: Fit for Frailty - consensus best practice guidance for the care of older people living in community and outpatient settings - a report from the British Geriatrics Society. 2014. https://www.bgs.org.uk/sites/default/files/content/resources/files/2018-05-23/fff_full.pdf. Accessed 14 Oct 2022.

[29] Hyde M, Higgs P, Wiggins RD, Blane D (2015). A decade of research using the CASP scale: key findings and future directions. Aging Ment Health.

[30] Hyde M, Wiggins RD, Higgs P, Blane DB (2003). A measure of quality of life in early old age: the theory, development and properties of a needs satisfaction model (CASP-19). Aging Ment Health.

[31] Price AJ, Crampin AC, Amberbir A, Kayuni-Chihana N, Musicha C, Tafatatha T, Branson K, Lawlor DA, Mwaiyeghele E, Nkhwazi L (2018). Prevalence of obesity, hypertension, and diabetes, and cascade of care in sub-Saharan Africa: a cross-sectional, population-based study in rural and urban Malawi. Lancet Diabetes Endocrinol.

[32] Vellakkal S, Millett C, Basu S, Khan Z, Aitsi-Selmi A, Stuckler D, Ebrahim S (2015). Are estimates of socioeconomic inequalities in chronic disease artefactually narrowed by self-reported measures of prevalence in low-income and middle-income countries? Findings from the WHO-SAGE survey. J Epidemiol Community Health.

[33] Tanzania in figures 2018. The United Republic of Tanzania National Bureau of Statistics. Dodoma. 2019. https://www.nbs.go.tz/nbs/takwimu/references/Tanzania_in_Figures_2018.pdf. Accessed 14 Oct 2022.

[34] Odland ML, Payne C, Witham MD, Siedner MJ, Barnighausen T, Bountogo M, Coulibaly B, Geldsetzer P, Harling G, Manne-Goehler J (2020). Epidemiology of multimorbidity in conditions of extreme poverty: a population-based study of older adults in rural Burkina Faso. BMJ Glob Health.

[35] Prados-Torres A, Calderon-Larranaga A, Hancco-Saavedra J, Poblador-Plou B, van den Akker M (2014). Multimorbidity patterns: a systematic review. J Clin Epidemiol.

[36] Stewart Williams J, Kowal P, Hestekin H, O’Driscoll T, Peltzer K, Yawson A, Biritwum R, Maximova T, Salinas Rodriguez A, Manrique Espinoza B, et al. Prevalence, risk factors and disability associated with fall-related injury in older adults in low- and middle-incomecountries: results from the WHO Study on global AGEing and adult health (SAGE). BMC Med. 2015;13:147. 10.1186/s12916-015-0390-8 PMC4495610 26099794

[37] Dotchin CL, Akinyemi RO, Gray WK, Walker RW (2013). Geriatric medicine: services and training in Africa. Age Ageing.

[38] Ministry of labour youth development and sports (2003). United Republic of Tanzania national ageing policy In.

[39] Osberg L, Mboghoina T. Social Protection of the Elderly in Tanzania: Current status and future possibilities. Research on Poverty Alleviation (REPOA). 2011; Brief number 24. Dar es Salaam. https://opendocs.ids.ac.uk/opendocs/handle/20.500.12413/1822. Accessed 14 Oct 2022.

[40] Counts CJ, Skordis-Worrall J (2016). Recognizing the importance of chronic disease in driving healthcare expenditure in Tanzania: analysis of panel data from 1991 to 2010. Health Policy Plan.

[41] Brinda EM, Andres AR, Enemark U (2014). Correlates of out-of-pocket and catastrophic health expenditures in Tanzania: results from a national household survey. BMC Int Health Hum Rights.

