# Supplementary material for: Multimorbidity and its socio-economic associations in community-dwelling older adults in rural Tanzania; a cross-sectional study
Source: BMC Public Health. 2022 Oct 14;22:1918. doi: 10.1186/s12889-022-14340-0 (PMC9569067; doi:10.1186/s12889-022-14340-0)
Supplement: Supplementary file 1 — Additional file 1: Table 1. List of conditions in the category ‘other clinical diagnoses’ by non-self-report. [file 12889_2022_14340_MOESM1_ESM.docx]

### Table 1 List of conditions in the category ‘other clinical diagnoses’ by non-self-report

| Clinical diagnosis | Frequency N=23 |
| --- | --- |
| Orthostatic hypotension | 4 |
| Goitre | 2 |
| Essential tremor | 2 |
| Old polio | 3 |
| Carpal tunnel syndrome | 1 |
| Chronic leg/pressure ulcer | 5 |
| Headache | 2 |
| Head trauma | 1 |
| Diabetic neuropathy | 1 |
| Bilateral lower limb paralysis following spinal infection | 1 |
| Blistering rash possible pemphigoid | 1 |
