# Supplementary material for: Multimorbidity and its socio-economic associations in community-dwelling older adults in rural Tanzania; a cross-sectional study
Source: BMC Public Health. 2022 Oct 14;22:1918. doi: 10.1186/s12889-022-14340-0 (PMC9569067; doi:10.1186/s12889-022-14340-0)
Supplement: Supplementary file 2 — Additional file 2: Table 2. Demographic/socio-economic characteristics of the sample by sex. [file 12889_2022_14340_MOESM2_ESM.docx]

### Table 2 Demographic/socio-economic characteristics of the sample by sex

| Demographic/health characteristic | Men N=99 | Women N=136 |
| --- | --- | --- |
| Continence problem: | 41 (41.4) | 55 (40.4) |
| Self-reported falls within 1 year:  None  One  Two or more | 73 (73.7)  11 (11.1)  15 (15.2) | 97 (71.3)  19 (14.0)  20 (14.7) |
| Self-reported hearing problem: | 34 (34.3) | 42 (30.9) |
| EURO-D depression  No depression  Depression ≥5/12 | 63 (63.6)  36 (36.4) | 63 (46.3)  73 (53.7) |
| IDEA cognitive test scores:  0-4 (poor cognitive function)  5-7 (moderate cognitive function)  8-12 (good cognitive function) | 9 (9.1)  17 (17.2)  73 (73.7) | 29 (21.3)  29 (21.3)  78 (57.4) |
| Measured high BP (N=234) | 44 (44.4) | 84 (62.2) |
