# Supplementary material for: Multimorbidity and its socio-economic associations in community-dwelling older adults in rural Tanzania; a cross-sectional study
Source: BMC Public Health. 2022 Oct 14;22:1918. doi: 10.1186/s12889-022-14340-0 (PMC9569067; doi:10.1186/s12889-022-14340-0)
Supplement: Supplementary file 3 — Additional file 3: Table 3. The prevalence of multimorbidity adjusted for frailty-weighting. [file 12889_2022_14340_MOESM3_ESM.docx]

### Table 3 The prevalence of multimorbidity adjusted for frailty-weighting

| Condition/type of multimorbidity | Self-report N from 235 (%) | Self-report adjusted  Prevalence (95% CI) | Clinical assessment N from 235 (%) | Clinical assessment adjusted  Prevalence (95% CI) |
| --- | --- | --- | --- | --- |
| Hypertension | 71 (30.5) | 25.44 (19.3-31.5) | 128 (54.5) | 48.14 (38.4-57.8) |
| Arthritis | 54 (23.0) | 22.83 (15.5-30.1) | 88 (37.4) | 34.45 (21.9-36.9) |
| Cataract | 21 (8.9) | 6.93 (2.7-11.0) | 36 (15.3) | 11.55 (6.3-16.7) |
| Dementia | 14 (6.0) | 3.33 (0.8-5.8) | 38 (16.2) | 4.95 (2.6-7.2) |
| Depression | 14 (6.0) | 7.05 (1.8-12.2) | 109 (46.4) | 39.06 (30.9-47.1) |
| GI | 21 (8.9) | 8.71 (5.4-11.9) | 40 (17.0) | 19.03 (12.1-25.9) |
| Respiratory | 17 (7.2) | 5.95 (-1.6-13.5) | 22 (9.4) | 8.80 (-0.1-17.7) |
| Stroke | 19 (8.1) | 3.40 (1.1-5.6) | 23 (9.8) | 5.55 (3.1-7.9) |
| Heart disease | 16 (6.8) | 4.92 (2.4-7.3) | 17 (7.2) | 8.35 (1.4-15.2) |
| Diabetes | 22 (9.4) | 7.56 (3.8-11.2) | 19 (8.1) | 5.62 (1.6-9.5) |
| HIV | 4 (1.7) | 2.33 (0.2-4.3) | 6 (2.6) | 2.53 (0.4-4.6) |
| TB | 2 (0.9) | - | 2 (0.9) | - |
| Urology | 5 (2.1) | - | 25 (10.6) | 8.9 (2.9-14.9) |
| Anaemia | 12 (5.1) | 4.24 (-1.2-9.7) | - | - |
| Epilepsy | 1 (0.4) | - | 1 (0.4) | - |
| Cancer | 2 (0.9) | - | 3 (1.3) | - |
| Other mental health | 5 (2.1) | - |  |  |
| Other CGA diagnoses | - | - | 23 (9.8) | 6.82 (3.3-10.4) |
| ≥2 falls | 35 (14.9) | 14.91 (7.8-21.9) | - | - |
| Continence problems | 96 (40.9) | 21.68 (13.8-29.5) | - | - |
| Hearing impairment | 76 (32.3) | 27.38 (18.4-36.2) | - | - |
| Multimorbidity | 77 (32.8) | 26.09 (16.7-35.4) | 174 (74.0) | 67.28 (57.0-77.5) |
| Mental health multimorbidity | 4 (1.7) | 0.57 (-0.4-1.5) | 26 (11.1) | 3.41 (2.2-4.5) |
| Non-communicable multimorbidity | 72 (30.6) | 23.02 (15.6-30.4) | 138 (58.7) | 49.50 (41.5-57.4) |
| Discordant multimorbidity | 27 (11.5) | 9.58 (3.2-16.0) | 120 (51.0) | 40.81 (34.2-47.5) |
| Geriatric multimorbidity | 118 (50.2) | 34.88 (29.3-40.5) |  | |
