# Supplementary material for: Multimorbidity and its socio-economic associations in community-dwelling older adults in rural Tanzania; a cross-sectional study
Source: BMC Public Health. 2022 Oct 14;22:1918. doi: 10.1186/s12889-022-14340-0 (PMC9569067; doi:10.1186/s12889-022-14340-0)
Supplement: Supplementary file 4 — Additional file 4: Table 4. The CASP-19 quality of life score by demographic and multimorbidity categories. [file 12889_2022_14340_MOESM4_ESM.docx]

### Table 4 The CASP-19 quality of life score by demographic and multimorbidity categories.

|  | Mean | Standard deviation | Test of statistical significance  P value |
| --- | --- | --- | --- |
| All (n=231) | 24.48 (range 0 – 53) | 11.63 | - |
| Men (n=98) | 22.38 | 12.55 |  |
| Women (n=133) | 26.03 | 10.68 | 0.021 |
| CGA not frail (n=143) | 19.03 | 9.32 |  |
| CGA frail (n=88) | 33.34 | 9.32 | <0.001 |
| Age 60-69 (n=87) | 19.53 | 10.16 |  |
| 70-79 (n=66) | 22.98 | 11.36 |  |
| 80 and over (n=78) | 31.27 | 10.17 | <0.001 |
| No self-reported multi-morbidity (n=156) | 21.85 | 11.62 |  |
| Self-reported multi-morbidity (n=75) | 27.87 | 10.97 | 0.002 |
| No non-self-reported multi-morbidity (n=60) | 16.95 | 9.49 |  |
| Non-self-reported multi-morbidity (n=171) | 27.12 | 11.17 | <0.001 |
| No geriatric multi-morbidity (n=116) | 19.31 | 10.22 |  |
| Geriatric multi-morbidity (n=115) | 29.70 | 10.62 | <0.001 |
