# Supplementary material for: Multimorbidity and its socio-economic associations in community-dwelling older adults in rural Tanzania; a cross-sectional study
Source: BMC Public Health. 2022 Oct 14;22:1918. doi: 10.1186/s12889-022-14340-0 (PMC9569067; doi:10.1186/s12889-022-14340-0)
Supplement: Supplementary file 5 — Additional file 5: Figure 1. The adjusted prevalence of multimorbidity/conditions by self- and non-self-reported methods. [file 12889_2022_14340_MOESM5_ESM.docx]

### Figure 1 The adjusted prevalence of multimorbidity/conditions by self- and non-self-reported methods
