# Supplementary material for: Multimorbidity and its socio-economic associations in community-dwelling older adults in rural Tanzania; a cross-sectional study
Source: BMC Public Health. 2022 Oct 14;22:1918. doi: 10.1186/s12889-022-14340-0 (PMC9569067; doi:10.1186/s12889-022-14340-0)

Figure 2 The overlap between self-reported multimorbidity, disability and CGA-diagnosed frailty


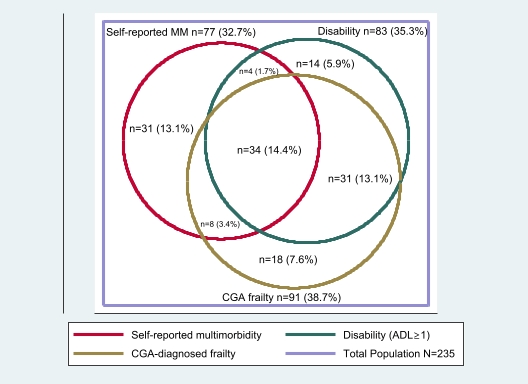

Supplement: Supplementary file 6 — Additional file 6: Figure 2. The overlap between self-reported multimorbidity, disability and CGA-diagnosed frailty. [file 12889_2022_14340_MOESM6_ESM.docx]
