# Supplementary material for: Multimorbidity and its socio-economic associations in community-dwelling older adults in rural Tanzania; a cross-sectional study
Source: BMC Public Health. 2022 Oct 14;22:1918. doi: 10.1186/s12889-022-14340-0 (PMC9569067; doi:10.1186/s12889-022-14340-0)

### Figure 3 The relationship between non-self-reported multimorbidity, CGA-diagnosed frailty and disability


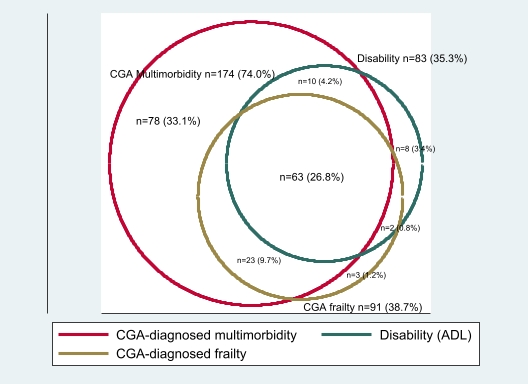

Supplement: Supplementary file 7 — Additional file 7: Figure 3. The relationship between non-self-reported multimorbidity, CGA-diagnosed frailty and disability. [file 12889_2022_14340_MOESM7_ESM.docx]
